# The phylogenetic position of the enigmatic Balkan *Aulopyge huegelii* (Teleostei: Cyprinidae) from the perspective of host-specific *Dactylogyrus* parasites (Monogenea), with a description of *Dactylogyrus omenti* n. sp.

**DOI:** 10.1186/s13071-017-2491-z

**Published:** 2017-11-03

**Authors:** Michal Benovics, Maria Lujza Kičinjaová, Andrea Šimková

**Affiliations:** Department of Botany and Zoology, Faculty of Science, Masaryk University, Kotlářská 2, 61137 Brno, Czech Republic

**Keywords:** Host specificity, Coevolution, Phylogeography, *Aulopyge*, Cyprininae, *Dactylogyrus*

## Abstract

**Background:**

The host specificity of fish parasites is considered a useful parasite characteristic with respect to understanding the biogeography of their fish hosts. *Dactylogyrus* Diesing, 1850 (Monogenea) includes common parasites of cyprinids exhibiting different degrees of host specificity, i.e. from strict specialism to generalism. The phylogenetic relationships and historical dispersions of several cyprinid lineages, including *Aulopyge huegelii* Heckel, 1843, are still unclear. Therefore, the aims of our study were to investigate (i) the *Dactylogyrus* spp. parasites of *A. huegelii*, and (ii) the phylogenetic relationships of *Dactylogyrus* spp. parasitizing *A. huegelii* as a possible tool for understanding the phylogenetic position of this fish species within the Cyprininae lineage.

**Results:**

Two species of *Dactylogyrus*, *D. vastator* Nybelin, 1924 and *D. omenti* n. sp., were collected from 14 specimens of *A. huegelii* from the Šujica River (Bosnia and Herzegovina). While *D. vastator* is a typical species parasitizing *Carassius* spp. and *Cyprinus carpio* L, *D. omenti* n. sp. is, according to phylogenetic reconstruction, closely related to *Dactylogyrus* species infecting European species of *Barbus* and *Luciobarbus*. The genetic distance revealed that the sequence for *D. vastator* from *A. huegelii* is identical with that for *D. vastator* from *Barbus plebejus* Bonaparte, 1839 (Italy) and *Carassius gibelio* (Bloch, 1782) (Croatia). *Dactylogyrus omenti* n. sp. was described as a species new to science.

**Conclusions:**

Our findings support the phylogenetic position of *A. huegelii* within the Cyprininae lineage and suggest that *A. huegelii* is phylogenetically closely related to *Barbus* and *Luciobarbus* species. The morphological similarity between *D. omenti* n. sp. and *Dactylogyrus* species of Middle Eastern *Barbus* suggests historical contact between cyprinid species recently living in allopatry and the possible diversification of *A. huegelii* from a common ancestor in this area. On other hand, the genetic similarity between *D. vastator* ex *A. huegelii* and *D. vastator* ex *C. gibelio* collected in Balkan Peninsula suggests that *A. huegelii* was secondarily parasitized by *D. vastator*, originating from *C. gibelio* after introduction of this fish species from Asia to Europe.

## Background

Parasites and their hosts are usually closely associated due to their coevolution, realized by reciprocal genetic adaptations between these interacting species. In evolutionary time, this leads to a selection for improvements in host–parasite recognition mechanisms [1]. The high degree of host specificity among parasites (generally, a parasite species is restricted to a single host species), reflecting parasite specialization, may arise from such coevolutionary interactions [2–4] In the case of high host specificity, the phylogeny of host-specific parasites may even follow the phylogeny and historical biogeography of their hosts as a result of co-speciation [5, 6]. However, parasite diversification can also be driven by host specialization following host switching resulting from strong ecological association, as was shown for monogeneans of marine fish [7]. The host specificity of freshwater fish parasites appears to be a useful characteristic in terms of understanding the biogeography of freshwater fishes (e.g. [8–11]). Basic host specificity is commonly expressed by the number of host species (also termed host range). However, other aspects, like the ecological performance of the parasite, the phylogenetic affinities of hosts, and the biogeographical distribution of the parasite, are important when expressing host specificity [12].

Gill ectoparasites of the genus *Dactylogyrus* Diesing, 1850 generally exhibit a high degree of host specificity and a high species diversity arising from the multitude of cyprinid fish species, which are common hosts of these parasite species [13]. Šimková et al. [14] defined several levels of host specificity for *Dactylogyrus* using an index of host specificity, expressed as the inverted value of the index of non-specificity proposed by Desdevises et al. [7]. Five *Dactylogyrus* groups were defined ranging from strict specialists, which occur on a single host species, to true generalists, which parasitize different, phylogenetically unrelated cyprinid host species. These host-specific parasites have a direct life-cycle, in which the larval stage (oncomiracidium) actively searches for suitable host species, using chemical cues for host recognition [15]. Therefore, among monogeneans, a high degree of adaptation to their host resource is required [16–19]. Several studies documented microhabitat restriction (i.e. preferred niche measured by specific gill positions) in *Dactylogyrus* species [20–24]. Since different parts of gills offer different types of substrate, niche preference is associated with a specific type and shape of attachment organ (haptor) in parasites assigned to *Dactylogyrus* [9, 21, 23]. Šimková et al. [23] also revealed that there is morphological adaptation of the haptor in species that specifically parasitize phylogenetically related hosts, such as *Dactylogyrus* species parasitizing *Cyprinus carpio* L. and *Carassius auratus* L. of the subfamily Cyprininae. The phylogeny of highly host-specific *Dactylogyrus* species reflects the biogeography and evolutionary history of their cyprinid hosts [25]. Besides some accidental infections of unsuitable hosts, the sharing of *Dactylogyrus* species among evolutionary divergent cyprinid species living in sympatry is rare [23].

The cyprinid fauna of the Balkan Peninsula is extremely rich in endemic species [26]. According to Oikonomou et al. [27], the Balkan freshwater fish fauna represents 59% of all known cyprinid species. The ancient Dessaretes lake system played an important role in cyprinid speciation, which originated during the Pleistocene and is considered as a hotspot of endemic freshwater biodiversity [28–32]. Presently, all the great lakes in the Balkan Peninsula, the Ohrid, Prespa, Mikri Prespa and Maliq lakes (the latter one was drained after World War II), are parts of this system. Albrecht & Wilke [30] also theorized that during the Miocene and Pliocene eras the whole Dessaretes basin was filled with water and that all lakes were connected. After the closing of the Korca Depression and connections between the Dessaretes and the Paratethys, the water level decreased and fragmentation of the populations triggered allopatric speciation, which led to rich freshwater fish diversity. Zardoya et al. [33] investigated the geographical origin of Balkan endemic cyprinids. They suggested that cyprinid fauna colonized the Balkan Peninsula during two different time periods. The first wave occurred during the Miocene and the second during the Plio-Pleistocene via river captures. The phylogenetic relationships among Balkan cyprinid taxa and their biogeographical histories have been actively studied over the last 25 years (e.g. [34–40]). Studying host-specific parasites, such as *Dactylogyrus*, may represent an additional tool for investigation and may shed more light on both the historical contacts between cyprinid hosts and their phylogeography.

The Dalmatian barbelgudgeon (*Aulopyge huegelii* Heckel, 1843), the only representative of the monotypic genus *Aulopyge*, is one of the many endangered cyprinid species of the Balkan Peninsula. Its distribution is limited to the Dinaric karst rivers and lakes of Croatia and Bosnia and Herzegovina [41–43]. Although previously quite abundant, in recent years *A*. *huegelii* populations have been declining [44]. Tsigenopoulos & Berrebi [43] considered the ancestor of *A. huegelii* as the first migration wave of cyprinids to the Mediterranean region, which found refuge in Dalmatia. According to the molecular clock, they estimated that European *Barbus* and *A. huegelii* diverged during the middle Miocene, which concurs with the first wave colonizing Balkan Peninsula [33]. On the basis of mitochondrial cytochrome *b* sequence data, Tsingenopoulos et al. [45] suggested that *A. huegelii* is the sister clade to the clade including *Barbus + Luciobarbus* lineages. However, Yang et al. [46] showed that *Aulopyge* is the sister taxon to the the European *Barbus* lineage, well separated from the *Luciobarbus* lineage, and, according to Wang et al. [47], the European *Barbus* (sensu stricto) lineage and *A. huegelii* share a common ancestor (originating in the Qinghai-Tibetan Plateau region about 19.4–7.8 Mya) with the species of the Asian genera *Schizothorax* and *Cyprinion*.

Until now, only a very few endemic cyprinid species from the Balkan Peninsula have been investigated for parasites [48–54]. As previously shown by Šimková et al. [25], phylogenetic relationships between *Dactylogyrus* lineages can reflect cyprinid phylogeny. Thus, we hypothesized that the phylogenetic relationships between host-specific *Dactylogyrus* species of *A. huegelii* and those parasitizing other closely related cyprinid species will support the phylogenetic position of this monotypic cyprinid genus. Therefore, the aims of our study were (i) to investigate the *Dactylogyrus* fauna of endemic *A. huegelii*, and (ii) to investigate the phylogenetic relationships between *Dactylogyrus* species parasitizing *A. huegelii* and those parasitizing species of the Cyprininae distributed in Europe, i.e. *Barbus* spp., *Carassius* spp. and *C. carpio* (the last two originating from Asia and widely distributed throughout the whole of Europe). As a result, we described a new species of *Dactylogyrus* collected from endemic *A. huegelii*.

## Methods

### Sampling and species identification

A total of 14 specimens of *Aulopyge huegelii* from the Šujica River, Bosnia and Herzegovina, were sampled in July 2015. Fish were dissected using standard methods [55]. *Dactylogyrus* specimens were collected from host gills, fins, head surfaces, and oral and nasal cavities, mounted on slides and covered with a mixture of glycerine and ammonium picrate (GAP [56]) for further identification. The identification of monogeneans was performed using Gussev [57] on the basis of the size and shape of the hard parts of the attachment organ, the haptor, and the reproductive organs which represent species-specific morphological characters. Identification to species level was performed using an Olympus BX51 microscope equipped with phase contrast optics. Several *Dactylogyrus* specimens were bisected; one half of the body (usually the half with the reproductive organs) was mounted on a slide for species identification, the other was individually preserved in 96% ethanol for DNA extraction. Basic epidemiological data, i.e. prevalence, mean abundance, minimum and maximum intensity of infection, were calculated for each species according to Bush et al. [58]. Prevalence, as the percentage of fish infected by a given parasite species, and mean abundance, as the mean number of parasite specimens per individual host taking into account both infected and uninfected hosts, were calculated.

### Morphometric data

Morphometric measurements of *Dactylogyrus* spp. specimens (modified according to Gussev [57]) were taken using Digital Image Analysis (Stream Motion). All measurements of morphometric characters are in micrometres and are presented as the range followed by the mean and the number of measured specimens (*n*) in parentheses. The numbering of marginal hook pairs for *Dactylogyrus* follows the recommendations by Mizzele [59]. After measuring morphometric characters, the specimens were removed from GAP and remounted in Canada balsam, according to Ergens [60], and deposited as type-specimens in the Helminthological Collection of the Institute of Parasitology, Biology Centre of the Academy of Sciences of the Czech Republic, in České Budějovice (IPCAS).

### DNA extraction, amplification and sequencing

Parasites were removed from storage ethanol and dried by means of a vacuum centrifuge. DNA extraction was performed using a standard protocol (DNeasy Blood & Tissue Kit, Qiagen, Hilden, Germany). Partial 18S rDNA and entire ITS1 regions were amplified using primers S1 (5′-ATT CCG ATA ACG AAC GAG ACT-3′) and IR8 (5′-GCT AGC TGC GTT CTT CAT CGA-3′) [61], which anneal to the 18S and 5.8S rDNA regions, respectively. Each amplification reaction for partial the 18S rDNA and ITS1 regions was performed in a final volume of 15 μl, containing 0.3 μl of Taq polymerase, 1.5 μl buffer, 0.9 μl MgCl_2_, 0.3 μl of dNTPs, 1.5 μl of each primer and 2.5 μl of pure DNA (20 ng/μl). PCR was carried out using the following steps: 2 min at 94 °C, followed by 40 cycles of 1 min at 94 °C, 1 min at 53 °C and 90 s at 72 °C, and 10 min of final elongation at 72 °C. Partial 28S rDNA was amplified using the forward primer C1 (5′-ACC CGC TGA ATT TAA GCA-3′) and the reverse primer D2 (5′-TGG TCC GTG TTT CAA GAC-3′) [62]. PCR followed the protocol included in Šimková et al. [14]. PCR products were checked on 1.5% agarose gels, purified by using an ExoSAP-IT kit (Ecoli, Bratislava, Slovakia), following the manufacturer’s protocol, and sequenced directly using the PCR primers and BigDye Terminator Cycle sequencing kit (Applied Biosystems, Pardubice, Czech Republic). Sequencing was carried out using an ABI 3130 Genetic Analyzer (Applied Biosystems). The newly generated sequences were deposited in the GenBank database and molecular vouchers (hologenophores, paragenophores [63]) were deposited in the Helminthological Collection of the Institute of Parasitology, Biology Centre of the Academy of Sciences of the Czech Republic, in České Budějovice (IPCAS).

### Phylogenetic analyses

DNA sequences were aligned using fast Fourier transform in MAFFT [64]. To match the lengths of the newly obtained sequences to the sequences obtained from GenBank, they were optimized manually. A test of homogeneity to examine the congruence of two datasets (partial 18S with the ITS1 region vs 28S rDNA) was performed in PAUP* 4b10 [65]. Since the difference was not statistically significant (*P* = 0.737), the concatenated data were used for further phylogenetic analyses. The sequences of *Dactylogyrus extensus* Mueller & Van Cleave, 1932 parasitizing *C. carpio* were acquired from GenBank (accession numbers KM277459 and AY553629 for partial 18S rDNA with the ITS1 region and partial 28S rDNA sequences, respectively). The sequences of partial 18S rDNA with the ITS1 region and partial 28S rDNA for *Dactylogyrus vastator* Nybelin, 1924 and *Dactylogyrus anchoratus* (Dujardin, 1845) parasitizing *Carassius gibelio* (Bloch, 1782), and *Dactylogyrus* species parasitizing *Barbus barbus* L., *B. balcanicus* Kotlík, Tsigenopoulos, Ráb & Berrebi, 2002, *B. prespensis* Karaman, 1924, *Luciobarbus graecus* (Steindachner, 1895) and *L. albanicus* (Steindachner, 1870) (including Balkan endemic and non-endemic *Dactylogyrus* species) were included in phylogenetic analyses due to the proposed evolutionary proximity of the host species. The final tree was rooted using *Dactylogyrus* species of *C. gibelio* and *C. carpio* as the outgroup taxa, following Šimková et al. [23].

To analyze the genetic distances between the specimens of *D. vastator* from different host species, sequences of partial 18S rDNA and complete ITS1 available for *D. vastator* were obtained from GenBank. The uncorrected p-distances between *D. vastator* collected from 5 different host species from 7 localities were calculated using MEGA6 [66].

Gaps and ambiguously aligned regions were removed from the alignment using GBlocks v. 0.91 [67]. The most appropriate DNA evolution model was determined using the Akaike information criterion (AIC) in JModelTest 2.1.10 [68, 69]. Phylogenetic trees were inferred by Bayesian inference (BI) and Maximum Likelihood (ML) analyses using MrBayes 3.2 [70] and PhyML 3.0 [71], respectively. The search for the best ML tree was performed using NNI (nearest neighbour interchange) and SPR (subtree pruning and regrafting) branch swapping algorithms with six substitution categories. The clade support for ML was assessed by 1000 bootstrap pseudoreplicates. Bayesian inference trees were constructed using the MC^3^ algorithm with two parallel runs containing one cold and three hot chains. The analysis ran for 10^7^ generations and tree topologies were sampled every 100 generations. The first 25% of all saved trees were discarded as relative ‘burn-in’ periods according to standard deviation split frequencies (< 0.01). Posterior probabilities were calculated as the frequency of samples recovering any particular clade.

## Results

### Parasites of *A. huegelii*

All 14 dissected fish specimens were infected with monogenean parasites. *Dactylogyrus* spp. reached 93% prevalence in *A. huegelii* and represented two species. The first was *Dactylogyrus vastator*, a common parasite of *Carassius* spp. and *C. carpio*, and which also accidentaly infects some other fish species ([13], M. Benovics, unpublished data). Morphological identification confirmed that specimens of *D. vastator* from *A. huegelii* possess the same morphology of the hard parts of the haptor and reproductive organs (i.e. the shape was identical and the size of these parts was within the range of sizes included in original description of *D. vastator*). The second species is here described as *Dactylogyrus omenti* n. sp., which was not found on other endemic *Barbus* species, or any other cyprinids collected in the Balkan Peninsula, and is most likely specific to *A. huegelii*. Both *Dactylogyrus* species differed in their epidemiological characteristics (Table 1). The prevalence of *D. omenti* n. sp. was significantly higher than that of *D. vastator* (Fisher’s exact test, *P* = 0.006, *df* = 1). The abundance of *D. omenti* n. sp. was higher than that of *D. vastator* (Mann-Whitney test, *U*_(14)_ = 15.00, *Z* = 3.79, *P* < 0.001).

**Table 1:** Basic epidemiological data for *Dactylogyrus* species collected from *A. huegelii*

Species	P (%)	MA	I
*D. vastator*	29	0.3	1
*D. omenti* n. sp.	93	3.4	1–8

*Abbreviations*: *P*, prevalence; *MA*, mean abundance, *I*, intensity of infection

### Phylogenetic position of *Dactylogyrus* spp. parasitizing *A. huegelii*

**Fig. 1:**
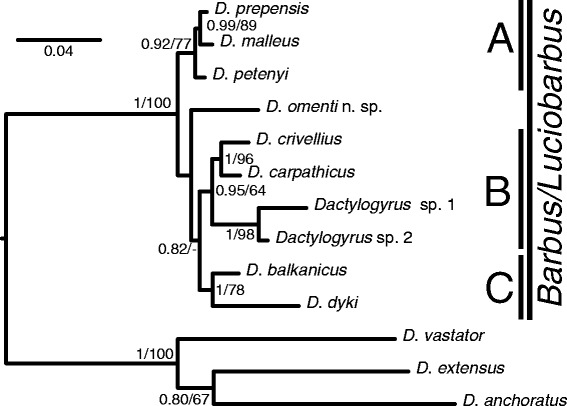
Phylogenetic tree constructed by Bayesian Inference (BI) analysis. The tree is based on concatenated data of partial 18S and ITS1 rDNA sequences with partial 28S rDNA sequences for selected *Dactylogyrus* species. Values along branches indicate BI posterior probabilities and Maximum Likelihood (ML) bootstrap values as BI/ML. Values < 0.80 for BI and < 50% for ML are indicated by dashes or not shown. Length of branches corresponds to the expected number of substitutions per site. Groups A, B and C refer to the different lineages of *Dactylogyrus* species parasitizing European *Barbus* and *Luciobarbus* species

A final concatenated sequence alignment was constructed using 1625 unambiguously aligned nucleotide positions. GTR + I + G was selected as the optimal evolution model. ML and BI analyses provided phylogenetic trees with similar topologies. The BI tree is presented in Fig. 1, where bootstrap values resulting from ML analysis and posterior probabilities resulting from BI analysis are included. Collection localities and GenBank accession numbers of all newly generated sequences used in the phylogenetic reconstructions are provided in Table 2.

**Table 2:** List of newly obtained *Dactylogyrus* species used for molecular analyses and phylogenetic reconstruction

Host species	*Dactylogyrus* spp.	Country	Locality	Coordinates	GenBank accession numbers	
					18S rDNA + ITS1 + 5.8S rDNA	28S rDNA
*Aulopyge huegelii*	*D. omenti* n. sp.	Bosnia and Herzegovina	Šujica, Duvansko polje	43°42′05.7″N, 17°15′50.5″E	KY201091	KY201105
	*D. vastator*				KY201092	KY201106
*Barbus balcanicus*	*D. petenyi*	Greece	Vardar, Axiopolis	40°59′28.4″N, 22°33′14.5″E	KY201097	KY201113
*Barbus barbus*	*D. carpathicus*	Czech Republic	River Svratka	49°05′32.1″N, 16°37′11.0″E	KY201098	KY201111
	*D. malleus*				KY201099	KY201112
*Barbus prespensis*	*D. crivellius*	Albania	Shkumbini, Perrenjas	41°03′50.9″N, 20°33′56.6″E	KY201094	KY201108
	*D. prespensis*				KY201096	KY201110
	*D. balkanicus*				KY201093	KY201107
	*D. dyki*	Greece	Aoos, Kalithea	40°01′16.7″N, 20°41′40.2″E	KY859804	KY859803
*Barbus plebejus*	*D. vastator*	Italy	River Po	na	KY201104	na
*Carassius gibelio*	*D. vastator*	Czech Republic	River Dyje	48°48′09.4″N, 16°50′19.3″E	KY201103	na
	*D. anchoratus*				KY859795	KY863555
	*D. vastator*	Croatia	Baštica Reservoir	44°11′34.1″N, 15°24′40.7″E	KY207446	na
*Luciobarbus albanicus*	*Dactylogyrus* sp. 1	Greece	Lake Trichonis, Panetolio	38°35′20.2″N, 21°28′02.7″E	KY201100	KY201114
*Luciobarbus graecus*	*Dactylogyrus* sp. 2	Greece	Sperchios, Ypati	38°54′14.3″N, 22°17′30.2″E	KY201101	KY201115

*Abbreviation*: *na*, not available

The resulting tree for *Dactylogyrus* spp. supports the close phylogenetic relationship of *A. huegelii* to endemic Mediterranean *Barbus* and *Luciobarbus* species and to the widely distributed European *Barbus barbus*, as previously shown by molecular phylogenetic studies of cyprinid fishes [43, 45, 47, 72], i.e. *Dactylogyrus omenti* n. sp. from *A. huegelii* was nested within *Dactylogyrus* spp. from *Barbus* species. *Dactylogyrus vastator* clustered with *D. extensus* from *C. carpio* and with *D. anchoratus* from *C. gibelio*. This clade was well separated from the clade of *Dactylogyrus* species parasitizing *Barbus*, *Luciobarbus* and *A. huegelii*. By comparing the genetic distances of *D. vastator* specimens from different hosts using the sequences of partial 18S and the ITS1 regions (Table 3), we conclude that *D. vastator* from *A. huegelii* is genetically identical to *D. vastator* collected from *C. gibelio* from Croatia and *Barbus plebejus* Bonaparte, 1839 from Italy. In comparison to species collected in central Europe and eastern Asia, *D. vastator* from *A. huegelii* is closer to *D. vastator* of *C. carpio* (p-distance = 0.003) than to *D. vastator* of *C. gibelio* from the Czech Republic or to *D. vastator* of *C. auratus* from China (p-distance > 0.043).

**Table 3:** Uncorrected pairwise distances between sequences for *D. vastator* collected from *Aulopyge huegelii* and different species of Cyprininae

Host species^a^		Locality	2	3	4	5	6	7
1	*Aulopyge huegelii* (KY201091)	River Šujica, Bosnia and Herzegovina	0.000	0.001	0.042	0.042	0.047	0.004
2	*Barbus plebejus* (KY201104)	River Po, Italy		0.001	0.042	0.042	0.047	0.004
3	*Carassius gibelio* (KY207446)	Baštica, Croatia			0.043	0.043	0.048	0.003
4	*Carassius gibelio* (KY201103)	River Dyje, Czech Republic				0.002	0.004	0.047
5	*Carassius auratus* (KJ854363)	Nanyang, Henan, China					0.004	0.047
6	*Carassius auratus* (KM487695)	River Ergis, China						0.051
7	*Cyprinus carpio* (AJ564159)	River Morava, Czech Republic						

^a^GenBank accession numbers included

Genetic distances were calculated using the sequences of partial 18S rDNA and ITS1 (see Table 2 for accession numbers for *D. vastator* sequences generated in this study)

*Dactylogyrus* species recovered from *Barbus* spp. formed a paraphyletic group with the nested position of *Dactylogyrus* spp. from Greek *Luciobarbus* and *D. omenti* n. sp. Three well- (or moderately-) supported groups were recognized for *Dactylogyrus* species collected from *Barbus* and *Luciobarbus* hosts (Fig. 1). Group A (PP = 0.92, BS = 77) comprised *D. prespensis* Dupont & Lambert, 1986, *D. malleus* Linstow, 1877 and *D. petenyi* Kastak, 1957, which exhibit a similar shape of the male copulatory organ (MCO). Group B was formed by two well supported clades, the first including *D. carpathicus* Zachvatkin, 1951 and *D. crivellius* Dupont & Lambert, 1986 collected from *Barbus*, and the second including two undescribed species *Dactylogyrus* sp. 1 and *Dactylogyrus* sp. 2 collected from Greek *Luciobarbus*. All these species exhibit a similar shape of the haptoral hard parts, especially in having a cross-shaped connective ventral bar with 5 marginal extremities, but differ between clades in the shape of the MCO. The last supported grouping (group C in Fig. 1, PP = 1, BS = 78) comprised *D. balkanicus* Dupont & Lambert, 1986 and *D. dyki* Ergens & Lucky, 1959. While *D. dyki* is a widely distributed European species (i.e. infecting a wide range of *Barbus* spp.), *D. balkanicus* appears to be endemic to the Balkan Peninsula, and they both share a similar shape of the MCO. *Dactylogyrus omenti* n. sp. was found at the basal position in the group of *Dactylogyrus* species parasitizing *Barbus* and *Luciobarbus*. However, the phylogenetic position of *D. omenti* n. sp. in relation to *Dactylogyrus* groups A, B and C was not resolved.

**Family Dactylogyridae Bychowsky, 1933**

**Genus** **Dactylogyrus** **Diesing, 1850**

***Dactylogyrus omenti*** **n. sp.**

***Type-host*** **:** *Aulopyge huegelii* Heckel, 1843 (Cypriniformes: Cyprinidae).

***Type-locality*** **:** Locality Duvansko polje, River Šujica, Bosnia and Herzegovina (43°42′05.7″N, 17°15′50.5″E).

***Type-material*** **:** The holotype, 4 paratypes, 1 hologenophore and 3 paragenophores are deposited under the accession number IPCAS M-629.

***Site on host*** **:** Gill lamellae.

***Representative DNA sequences*** **:** A nucleotide sequence of partial 28S rDNA (791 bp long; KY201105) and nucleotide sequences representing a fragment (939 bp long; KY201091) including partial 18S rDNA (446 bp), the ITS1 region (493 bp) and 5.8S (6 bp). No intraspecific variability was found (6 specimens were analyzed).

***ZooBank registration*** **:** To comply with the regulations set out in article 8.5 of the amended 2012 version of the International Code of Zoological Nomenclature (ICZN) [73], details of the new species have been submitted to ZooBank. The Life Science Identifier (LSID) of the article is urn:lsid:zoobank.org:pub:723FC725-1C88-4DF6-8ECE-ADC1EE658F8B. The LSID for the new name *Dactylogyrus omenti* is urn:lsid:zoobank.org:act:697DD685-1B87-4FB4-B3CA-65000EC772FF.

***Etymology*** **:** The specific name is derived from Latin (*omentum* = membrane, bowels) and refers to the shape of the accessory piece.

### Description

**Fig. 2:**
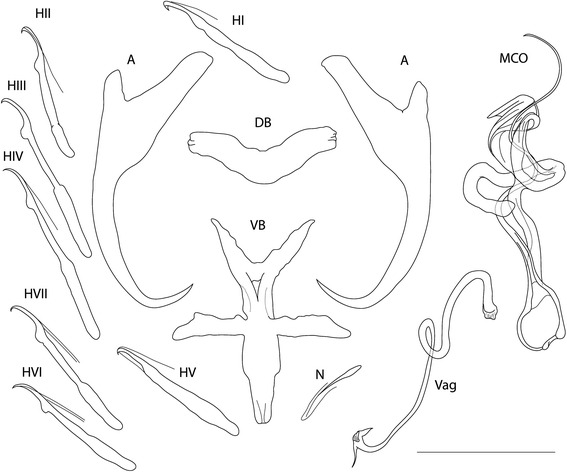
Drawings of hard parts of haptor and reproductive organs of *Dactylogyrus omenti* n. sp. *Abbreviations*: A, anchors; DB, dorsal connective bar; VB, ventral connective bar; H, marginal hooks (pairs I–VII); N, needle; MCO, male copulatory organ; Vag, vagina. *Scale-bar*: 20 μm

**Fig. 3:**
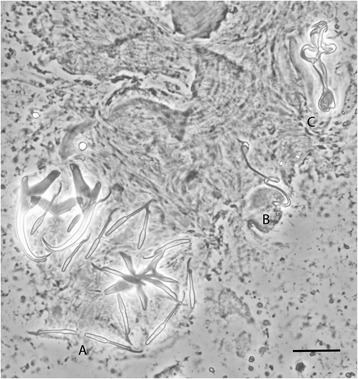
Phase contrast photomicrograph of hard parts of *Dactylogyrus omenti* n. sp. *Abbreviations*: A, haptor; B, vagina; C, male copulatory organ. *Scale-bar*: 20 μm

[Based on 13 specimens in GAP; Figs. 2 and 3.] Body length 230–522 (362; *n* = 3), with greatest width 57–128 (95; *n* = 3), usually near mid-length. One pair of anchors (dorsal), inner length 37–41 (38; *n* = 10), outer length 34–37 (35; *n* = 10). Inner root long, extending to broader base in its medial part, 11–16 (14; *n* = 10); outer root short, slightly pointed outward, 3–6 (5; *n* = 10), with moderately curved shaft and short turned-in point, 6–7 (6; *n* = 10). Dorsal bar saddle-shaped, with subterminal folding, total length 21–23 (22; *n* = 10), total width 4–5 (4; *n =* 10). Ventral bar airplane shaped, five-pointed, total length 28–31 (30; *n* = 10), total width 22–27 (24; *n* = 10); Marginal hooks 7 pairs, dissimilar in size, each with delicate point, long shaft with expanded proximal subunit; filament loop partial, reaching close to level of expanding part of shaft. Hook lengths (*n* = 10): pair I 21–22 (21), pair II 19–24 (21), pair III 22–26 (25), pair IV 25–32 (28), pair V 21–23 (22), pair VI 20–24 (22), pair VII 22–28 (24). One pair of needles located near marginal hooks of pair V, length 11–12 (12; *n* = 10). Vagina sclerotized, elongated, usually twisted tube, with anchor shaped opening (opens dextrally), trace length 54–62 (56; *n* = 10). MCO comprising basally articulated copulatory tube and accessory piece, total length 34–38 (36; *n* = 10). Copulatory tube delicate, undulated in its medial part, distally narrowing to non-enveloped termination, tube-trace length 45–54 (49; *n* = 10); with thick-walled base, length 8–10 (8; *n* = 10), width 6–7 (6; *n* = 10). Accessory piece passing to colon-shaped process encircling medial part of copulatory tube, in distal portion and shield-like membranous broadening supporting copulatory tube.

### Remarks

According to the morphology of the haptoral hard parts and reproductive organs, *D*. *omenti* n. sp. is most similar to *Dactylogyrus affinis* Bychowsky, 1933 (recorded from *Barbus lacerta* Heckel, 1843 [74], *Luciobarbus brachycephalus* (Kessler, 1872) [75], *L. capito* (Güldenstädt, 1773) [76] and *L. xanthopterus* Heckel, 1843 [77]), *Dactylogyrus deziensioides* Gussev, Jalali & Molnar, 1993 (from *L. kersin* Heckel, 1843 [78]), and *Dactylogyrus crivellius* (from *B. prespensis*) [48, 79]. However, *D. omenti* n. sp. differs from these species by the size of its haptoral hard parts, which are smaller (comparative morphometric data are provided in Table 4). In general, the configuration of hard haptoral elements and the shape of the ventral bars also resembles *Dactylogyrus* spp. from Moroccan *Luciobarbus* spp. described by el Gharbi et al. [80]. The MCO of *D. omenti* n. sp. most closely resembles the MCO of *D. deziensioides*, due to the presence of the colon-shaped process of the accessory piece encircling the copulatory tube. However, the copulatory tube of *D. deziensioides* is massive and short, in contrast with the delicate and long copulatory tube of *D. omenti* n. sp. In the original description of *D. affinis*, Bychowsky [81] pointed out the poor visibility of the end of copulatory tube, because of a saucer-shaped broadening of the accessory piece. This observation corresponds with the poor visibility of the medial part of the copulatory tube of *D. omenti* n. sp., on account of the shield-like broadening. Nevertheless, the colon-shaped process of the accessory piece is missing in the original drawing of *D. affinis*. The elongated twisted vagina of *D. affinis* markedly resembles the shape of the vagina of *D. omenti* n. sp*.* In regards to *D. crivellius*, *D. omenti* n. sp. differs in having a longer copulatory tube, larger colon-shaped part of the accesory piece and a thinner and longer vagina.

**Table 4:** Comparative metrical data (in μm) for hard parts of the haptor and reproductive organs of *D. omenti* n. sp. and morphologically similar *Dactylogyrus* spp.

Character		*D. omenti* n. sp.	*D. affinis*	*D. deziensioides*	*D. crivellius*
Body	length	230–522	600^a^	470^a^	–
	width	57–128	160^a^	120^a^	–
Anchors	inner length	37–41	46–65	47–49	58–61
	outer length	34–37	39–50	35–37	49–52
	inner root length	11–16	12–21	16–17	19–20
	outer root length	3–6	3–6	5–6	7–8
	point length	6–7	12–15	12–14	17–18
Ventral bar	length	28–31	50^a^	43–47	42^a^
	width	22–27	34^a^	30–32	26^a^
Dorsal bar	length	21–23	36–46	33^a^	42–43
	width	4–5	4–8	3–4	9^a^
Marginal hooks	length	19–32	21–33	25–28	31–34
Needle	length	11–12	–	–	–
MCO	length	34–38	37–47	46^a^	58–62
Vagina	length	54–62	40–50	–	–

^a^Maximum values of measured trait

Measurements of *D. affinis, D. deziensioides* and *D. crivellius* are obtained from [91]

## Discussion

With two species now known, the overall species richness of *Dactylogyrus* from *A. huegelii* is similar to that of other *Barbus* species from southern (France and Spain) and central Europe, for which 1–3 *Dactylogyrus* species per host species have been documented [25, 82]. The species richness of *Dactylogyrus* from *Barbus* species in the Balkan Peninsula ranges between 1 and 5 *Dactylogyrus* species per host species [e.g. 48]. While endemic and widely distributed *Barbus* species share several *Dactylogyrus* species (such as *D. dyki*, *D. petenyi*, *D. crivellius*, *D. carpathicus*, *D. malleus* and *D. balkanicus*), *D. omenti* n. sp. was recognized only from *A. huegelii* in this study, and therefore it is likely specific for this cyprinid species.

*Dactylogyrus vastator*, the parasite species with a large body size, has been widely reported from wild and farmed populations of *C. carpio* and *Carassius* spp., both of which belong to the subfamily Cyprininae (e.g. [52, 83–86]). In addition, the accidental infection of *D. vastator* was also found on some other cyprinid species (especially on *Barbus* [13], M. Benovics, unpublished data). Our study revealed a moderate prevalence of *D. vastator* on *A. huegelii*, which indicates that the infection of *D. vastator* on this endemic cyprinid species is not an accident. However, the low parasite infrapopulation size may indicate that this host is probably not suitable for maintaining parasite populations. *Cyprinus carpio* and *C. gibelio* may harbour up to nine different *Dactylogyrus* species [25, 87, 88]. The presence of only *D. vastator* on *A. huegelii* from this wide range of *Dactylogyrus* species could indicate: (i) the absence of other *Dactylogyrus* spp. on *C. carpio* and *Carassius* species potentially living in sympatry with *A. huegelii*; (ii) strict host specificity among other *Dactylogyrus* spp. of *C. carpio* and *C. gibelio* resulting from reciprocal coadaptation; or (iii) different morphologies of gill filaments providing microhabitats suitable for some *Dactylogyrus* species (i.e. large species such as *D. vastator* or *D. extensus*), but unsuitable for others (i.e. small species such as *D. achmerowi* Gussev, 1955, *D. falciformis* Akhmerov, 1952 or *D. minutus* Kulwiec, 1927). To test these hypotheses, further investigation of parasite communities on *C. carpio* and *Carassius* spp. potentially living in sympatry with *A. huegelli* and analyses of the niche preferences of *Dactylogyrus* parasites (i.e. the preferred positions on fish gills) are necessary. *Dactylogyrus vastator* usually infects small fingerlings, where overpopulation may result in the mortality of the host. According to Uspenskaya [89], 40 specimens of *D. vastator* could possibly cause the death of a fish with a body length of 2 cm. This is not the case with *A. huegelii*, where very low abundance was found, i.e. only a single specimen of *D. vastator* per individual fish, suggesting that mortality of this host is unlikely. This low abundance is conflicting with optimal conditions for the development of this parasite species [90, 91], because high population growth and consequently a high intensity of infection on the part of *D. vastator* are expected in southern regions, which have high water temperatures in summer. Possible explanations could be that the mobility of *D. vastator* larvae is restricted by different suboptimal environmental factors, resulting from the habitat preference of *A. huegelii*; that is, finding new hosts in these conditions may be more difficult. Alternatively, this species could be competitively excluded by higher populations of the second host-specific species parasitizing *A. huegelii*, *Dactylogyrus omenti* n. sp. [79, 92], which, in our study, was the most abundant *Dactylogyrus* species on *A. huegelli*.

We hypothesized that *Dactylogyrus* species are a good indicator of evolutionary relationships between cyprinid host species. Despite the low abundance of *D. vastator* on *A. huegelii*, this record supports the phylogenetic relationships of *A. huegelii* to species of the Cyprininae originating from Asia and probably introduced into Europe, i.e. *C. carpio* and *Carassius* spp. This parasite species was also found in very low abundance (1 specimen per fish and a prevalence of 20%) on *Barbus plebejus* during our field study in Italy. *Aulopyge huegelii* possibly offers a similar type of substrate, which, in the case of *Dactylogyrus* spp., is gill filaments, and, therefore, common *Dactylogyrus* spp. parasitizing *C. carpio* and *Carassius* species [83, 85, 88, 93] can also develop and inhabit closely phylogenetically related species such as *A. huegelii* and some *Barbus* species. This may support the finding of Shamsi et al. [88] indicating that the transmission of *D. anchoratus* from common carp to *Barbus sharpeyi*, an important native fish species, takes place despite the high host specificity of many *Dactylogyrus* species. Šimková et al. [25] proposed that the phylogeny of *Dactylogyrus* reflects, at least partially, the phylogeny of their cyprinid host species (depending more or less on the level of host specificity of particular species). According to Kohlmann et al. [94], European and Asian cyprinids share a common ancestor from central Eurasia. While *C. carpio* is widely distributed in the Eurasian region, species of the *C. auratus* complex are native to eastern Asia and were only recently imported into Europe and other continents [26, 95]. There are no paleontological records of the *C. auratus* complex in Europe before the Pleistocene [95]. By computing pairwise genetic distances between *D. vastator* from different host species, we showed that *D. vastator* of *A. huegelii* collected in Bosnia and Herzegovina was genetically identical with *D. vastator* of *C. gibelio* from Croatia and *Barbus plebejus* collected in Italy. Moreover, this form of *D. vastator* appears to be evolutionarily closer to *D. vastator* collected from *C. carpio* than to *D. vastator* from *C. auratus* and *C. gibelio* from central Europe*.* However, as we have only limited data on the distribution of *D. vastator* in *C. carpio* or *Carassius* spp., and no data on the distribution and origin of these fish species in Mediterranean areas (the Apennine and Balkan Peninsulas), this may indicate two scenarios of historical dispersion of *D. vastator*: (i) *D. vastator* occurring in endemic Mediterranean fishes originated from the historical dispersion of *C. carpio* to the Mediterranean Peninsulas, where former population of *D. vastator* parasitizing non-native *C. carpio* switched to phylogenetically related Mediterranean cyprinid species and introduced *C. gibelio*, and then slightly genetically differentiated from the former population; (ii) Genetic differentiation took place among geographically isolated populations of *D. vastator* parasitizing *C. carpio* and the representatives of *C. auratus* complex, and the genetically differentiated form of *D. vastator* was, with their non-native hosts (probably with *C. gibelio*), introduced more recently to different Mediterranean Peninsulas and switched to phylogenetically related endemic Mediterranean cyprinids. Both scenarios may suggest the potential risk of *D. vastator* infection for endemic cyprinids. Data on the infection levels of *D. vastator* in non-native *C. carpio* and *C. gibelio* in Mediterranean areas may be helpful to clarify whether endemic cyprinids serve as real or accidental host species for this species. Unfortunately, such data are not at disposal in this study.

The phylogenetic position of *D. omenti* n. sp. was found to be nested within *Dactylogyrus* of *Barbus* and *Luciobarbus*. The morphological similarity between the copulatory organs and haptoral hard parts of *D. omenti* n. sp. and *D. affinis* and *D. deziensioides* indicates the potentially earlier diversion of the newly described species from species parasitizing *Barbus* and *Luciobarbus* species from Kazakhstan, Turkey and Middle East. This supports the close phylogenetic affinity of *A. huegelii* with ancestral *Barbus* lineages of Asia, from which *A. huegelii* and European *Barbus* lineages supposedly emerged [43]. Unfortunately, the lack of molecular data for *D. affinis* and *D. deziensioides* makes further examination of evolutionary connections currently impossible. With the shape of its haptoral hard elements, especially its typical cross-shaped ventral bar with five extremities, *D. omenti* n. sp. resembles *Dactylogyrus* of Greek and Moroccan *Luciobarbus* (see [80] for their morphology) and also *D. carpathicus* and *D. crivellius* from widely distributed *Barbus* species [48, 82]. It was suggested that the shape of the haptoral hard parts appears to be more suitable for resolving phylogenetic relationships between lineages of a given monogenean genus, while the shape of the reproductive organs is more suitable for identification at the species level because of its faster evolutionary change [23, 96–99]. This may indicate that *D. omenti* n. sp. is evolutionarily closer to the earlier mentioned species than to other *Dactylogyrus* of *Barbus*, possessing a different type of ventral bar. Nevertheless, our results showed that four *Dactylogyrus* spp. with a cross-shaped ventral bar with 5 extremities, i.e. *D. crivellius*, *D. carpathicus*, *Dactylogyrus* sp. 1 and *Dactylogyrus* sp. 2 (clade B in Fig. 1), formed a well supported (PP = 0.95, BS = 64) monophyletic group to the exclusion of *D. omenti* n. sp. The unexpected phylogenetic position of *D. omenti* n. sp. indicates that using only the shape of the haptor as a marker for solving phylogenetic relationships in monogenean species with rapid diversification is not advisable and that the shape of the reproductive organs should be taken into account. However, the phylogenetic relationships between other *Dactylogyrus* species included in our phylogenetic reconstruction follow haptor morphology, specifically the shape of the connective bars and hooks. This is true of the monophyletic group of *D. balkanicus* and *D. dyki* (group C), which possess a similar shape of hard parts of attachment organ (anchors, connective bars and marginal hooks) [48], though the two species vary in the dimensions of their haptoral hard parts [79]. Additionally, the copulatory organ of both species is similar. The fast development of variations in reproduction organs is considered as a mechanism for avoiding hybridization in the case of multiple congeneric monogenean species living in overlapping microhabitats [100]. This is also true for *Dactylogyrus* species parasitizing *Barbus*. Of a possible seven *Dactylogyrus* spp., *Barbus* and *Luciobarbus* species usually harbour only *Dactylogyrus* species with copulatory organs of a markedly different shape, representing different phylogenetic lineages ([80, 92, 101]. For instance, as is shown in the present study, *B. prespensis* hosts four species with differently shaped copulatory organs, *D. balkanicus*, *D. crivellius*, *D. dyki* and *D. prespensis*, representing three different phylogenetic lineages (see Table 2 and Fig. 1). Also the morphologically similar and phylogenetically close species, such as *D. dyki* and *D. balkanicus*, were not present on the same host species in one population.

## Conclusions

*Dactylogylrus omenti* n. sp. was recognized as a potentially strict specialist of *A. huegelii*. Concluding from the expected high degree of host specificity of *Dactylogyrus* parasites and presence of *D. vastator*, a typical parasite of *C. carpio* and *Carassius* spp., on *A. huegelii*, or the phylogenetic position of *D. omenti* n. sp., the *A. huegelii* is a taxon closely related to European *Barbus* and *Luciobarbus* and to the Cyprininae of Asian origin. Regarding hard morphological characters, *D. omenti* n. sp. resembles species of *Dactylogyrus* parasitizing species of *Barbus* and *Luciobarbus* from the Middle East and Kazakhstan. Similarities in the shape of hard parts may suggest the origin of *D. omenti* n. sp. in this region and also an evolutionary proximity of endemic Cyprininae from the Middle East and Kazakhstan to *A. huegelii*. The genetic distances between *D. vastator* collected from different host species revealed that *D. vastator* in *A. huegelii* is identical with *D. vastator* of Balkan *C. gibelio* and closer to the central European *C. carpio* rather than to *C. auratus* complex. These results are indicating recent host switch of *D. vastator* between different hosts in Europe. The phylogenetic reconstruction of *Dactylogyrus* species parasitizing different endemic *Barbus* spp. and *Luciobarbus* spp. in the Balkan Peninsula and widely distributed European *Barbus* spp. revealed that, despite the generally accepted view that the morphology of the attachment organ is the best tool for resolving phylogenetic relations (based on morphological characters only) between *Dactylogyrus* species, the shape and size of the copulatory organs of rapidly evolving monogeneans have to be taken into consideration. But most importantly, only the combination of both morphological characters together with molecular data should be used for resolving the phylogeny and detection of potentially hidden diversity.

## Abbreviations

BI: Bayesian inference analysis
BS: Bootstrap values resulting from maximum likelihood analysis
GAP: Mixture of glycerine and ammonium picrate
MC^3^: Metropolis-coupled Markov chain Monte Carlo analysis
MCO: Male copulatory organ
ML: Maximum likelihood analysis
PP: Posterior probability resulting from Bayesian inference analysis
